# MicroRNA-153-3p enhances cell radiosensitivity by targeting BCL2 in human glioma

**DOI:** 10.1186/s40659-018-0203-6

**Published:** 2018-12-11

**Authors:** Deyu Sun, Yi Mu, Haozhe Piao

**Affiliations:** 1 Radiation Oncology Department of Gastrointestinal & Urinary & Musculoskeletal Cancer, Liaoning Cancer Hospital & Institute, Shenyang, Liaoning China; 2 Department of Neurosurgery, Liaoning Cancer Hospital & Institute, No.44 Xiao Heyan Street, Shenyang, 110042 Liaoning China

**Keywords:** Glioma, Radiosensitivity, miR-153-3p, BCL2

## Abstract

**Background:**

Glioma is the most prevalent malignant tumor in human central nervous systems. Recently, the development of resistance to radiotherapy in glioma patients markedly vitiates the therapy outcome. MiR-153-3p has been reported to be closely correlated with tumor progression, but its effect and molecular mechanism underlying radioresistance remains unclear in glioma.

**Methods:**

The expression of miR-153-3p was determined in radioresistant glioma clinical specimens as well as glioma cell lines exposed to irradiation (IR) using quantitative real-time PCR. Cell viability, proliferation and apoptosis were then evaluated by MTT assay, colony formation assay, Flow cytometry analysis and caspase-3 activity assay in glioma cells (U87 and U251). Tumor forming was evaluated by nude mice model in vivo. TUNEL staining was used to detect cell apoptosis in nude mice model. The target genes of miR-153-3p were predicted and validated using integrated bioinformatics analysis and a luciferase reporter assay.

**Results:**

Here, we found that miR-153-3p was down-regulated in radioresistant glioma clinical specimens as well as glioma cell lines (U87 and U251) exposed to IR. Enhanced expression of miR-153-3p promoted the radiosensitivity, promoted apoptosis and elevated caspase-3 activity in glioma cells in vitro, as well as the radiosensitivity in U251 cell mouse xenografs in vivo. Mechanically, B cell lymphoma-2 gene (BCL2) was identified as the direct and functional target of miR-153-3p. Moreover, restoration of BCL2 expression reversed miR-153-3p-induced increase of radiosensitivity, apoptosis and caspase-3 activity in U251 cells in vitro. In addition, clinical data indicated that the expression of miR-153-3p was significantly negatively associated with BCL2 in radioresistance of glioma samples.

**Conclusions:**

Our findings suggest that miR-153-3p is a potential target to enhance the effect of radiosensitivity on glioma cells, thus representing a new potential therapeutic target for glioma.

## Background

Glioma is the one of most common primary malignancies that arises from glial or precursor cells occurring in brain and central nervous system [1]. These tumors exhibit extensive heterogeneity and consist of multiple different histological types, including anaplastic astrocytoma, glioblastoma multiforme and oligodendroglioma [2]. Until now, radiotherapy is synergistic with surgical and chemotherapy, which remains a major modality in the overall management of both early and advanced glioma therapy [3]. However, patients still have a highly aggressive clinical course and it is estimated the median survival of the Grade IV patients is only 12–15 months [4]. A major obstacle to such dismal prognosis is the common occurrence of radioresistance [5]. Hence, there is an urgent need to explore the molecular mechanisms responsible for the radioresistance of human glioma.

Recently, microRNAs (miRNAs) have gained significant interest in predicting and modifying radio- and chemotherapy in cancer research [6], which are members of a rapidly growing class of naturally occurring small (21~22 nt) non-coding RNAs [7]. They can mediate post-transcriptional gene silencing and regulate various pathophysiological processes including apoptosis, proliferation, differentiation, etc [7]. Moreover, abnormal expression of miRNAs are associated with the development and progression of cancer [8]. In recent years, the expression of several miRNAs has been changed in radioresistant cell lines. For example, miR-662 is upregulated in radioresistant colorectal cancer cells [9]. MiR-338-5p is strongly downregulated in esophageal squamous cell carcinoma (ESCC) cell lines (TE-4R) with acquired resistance to the irradiation (IR) treatment [10]. What’s more, miRNAs participate in regulating radiosensitivity by in different types of malignancies. MiRNA-203 induces nasopharyngeal carcinoma radiosensitivity through targeting IL-8/AKT signaling pathway [11]. MiR-106a confers an IR-resistant phenotype and implicated in prostate cancer progression [11]. Notably, miR-153-3p has been demonstrated to be low-expressed and function as a tumor suppressor in melanoma [12] and thyroid carcinoma [13]. Barciszewska et al. [14] manifested that miR-153-3p is lower expressed in glioblastoma compared with normal brain. Moreover, Chen et al. [15] revealed that miR-153-3p was correlated with radioresistant genes in non-small cell lung cancer when screening of miRNA profiles through GO analysis and pathway analysis. However, the roles and molecular mechanisms of miR-153-3p involved in radio-resistance and progression of glioma remain undefined.

BCL2 has a unique role as master negative regulator of apoptosis in mammalian cells [16]. The abnormal amplification of BCL2 protein has been reported in a wide range of malignancies, including leukemia, colorectal cancer, and lymphomas, and nervous system cancers [17–19]. Many independent studies, for example by ectopic expression or knockdown have demonstrated that upregulation of BCL2 abrogates apoptotic responses to radiotherapy, while downregulation of BCL2 leads to elevated sensitivity to IR [20]. Furthermore, BCL2 could be targeted by miR-16 and miR-181a involved in the tumorigenicity and radioresistance of glioma cells [21, 22]. Therefore, we hypothesized that aberrant expression of miR-153-3p might affect the radioresistance of glioma cells by regulating BCL2.

The current study aimed to determine whether miR-153-3p associated with radioresistance in glioma and reveal its biological function and the correlation with BCL2. Our investigations may help the clinicians select the best treatment strategy against glioma patients.

## Materials and methods

### Tissues samples

Glioma patients (n = 45, 34 male and 11 females, median age = 54 years, range, 40–64 years) were included into this study, who underwent surgery at the Liaoning Cancer Hospital & Institute (Liaoning, China) after histopathological confirmation (WHO criteria). These patients were treated with radiotherapy for over 6 months and classified into radiosensitive (n = 25) and radioresistant (n = 20) groups based on radiosensitivity index (RSI) values (RSI index > 0.5 means radioresistant) as previously reported [23]. The tumor tissues were sampled from contrast enhancing regions identified by intraoperative neuronavigation (Cranial Map Neuronavigation Cart 2, Stryker, Freiburg, Germany) during resection, which were then immediately frozen in liquid nitrogen and stored at − 80 °C until analysis. The present study was approved by the ethics committee of the Liaoning Cancer Hospital & Institute (Protocol 102563, Liaoning, China) and obtained the written informed consent from all patients.

### Cell culture and irradiation

Human glioma cell lines, U251 and U87 were purchased from the American Type Culture Collection (ATCC, Manassas, VA, USA) and cultured in Dulbecco’s modified Eagle’s medium (DMEM, Hyclone, MA, USA) supplemented with 10% FBS (Gibco, NY, USA) in a humidified incubator containing 5% CO_2_ at 37 °C. For IR, U251 and U87 cells were seeded in 100-mm culture dishes at a density of 1 × 10^6^ cells and cultured for 24 h, followed by exposed to laboratory X-ray generated by an irradiation apparatus (2100 C/D, VARIAN, CA, USA) with 0. 2. 4, 6 and 8 Gy at room temperature.

### Cell transfection

MiR-153-3p mimics (miR-153-3p) and its scrambled negative control (miR-NC) were synthesized by Genepharma Company (Shanghai, China). The full length sequences of BCL2 were amplified by PCR and sub-cloned into pcDNA3.1 vector (Invitrogen) to generate pcDNA-BCL2 (BCL2) plasmids. Before transfection, U251 and U87 cells were trypsinised and seeded onto 6-well plates to reach 70% cell confluence. Then, the transfection of miR-153-3p, miR-NC, BCL2 vector and empty vector was carried out at a final concentration of 100 nM using Lipofectamine 2000 (Invitrogen, California, USA) following the manufacturer’s procedure. The transfected cells were incubated for a further 48 h prior to IR.

### RNA extraction and quantitative real-time PCR (qRT-PCR)

Total RNA was extracted from tissues and cells using TRIzol (Invitrogen, Carlsbad, CA, USA). For the detection of miR-153-3p expression, cDNA synthesis was performed using TaqMan MicroRNA Array kit (Applied Biosystems, CA, USA). The relative expression level of miR-153-3p was determined by TaqMan Universal Master Mix II (Applied Biosystems) with U6 as the internal reference. For the detection of BCL2 mRNA expression, cDNA synthesis was performed by PrimeScript RT reagent kit (TaKaRa BIO, Shiga, Japan) with GAPDH as the normalization. The expression of BCL2 was measured using SYBR Premix Ex Taq™ II (TaKaRa BIO) reagent with GAPDH as the normalization. All qRT-PCR was performed in triplicate on ABI Prism 7900HT Real-Time PCR System (Thermo Fisher Scientific, Inc.). All primers were purchased from Guangzhou RiboBio Co., Ltd and listed as follows: miR-153-3p (NM_138303), 5′-ACACTCCAGCTGGGTTGCATAGTCACAAA-3′ (forward) and 5′-CAGTGCGTGTCGTGGAGT-3′ (reverse); U6 (NR_138085), 5′-CCCTTCGGGGACATCCGATA-3′ (forward) and 5′-TTTGTGCGTGTCATCCTTGC-3′ (reverse); BCL2 (NM_000657), 5′-GAACTGGGGGAGGATTGTGG-3′ (forward) and 5′-GCCGGTTCAGGTACTCAGTC-3′ (reverse); GAPDH (NM_002046), 5′-GGTGAAGGTCGGAGTCAACG-3′ (forward) and 5′-GCATCGCCCCACTTGATTTT-3′ (reverse). Data analysis was performed using the 2^−ΔΔCt^ method.

### Cell viability assay

After IR treatment, transfected cells were seeded in 96-well plates at a density of 4000 cells per well. To assess cell viability, the MTT assay was performed according to the manufacture’s instruction. In brief, each well was added 10 μl of 5 mg/ml MTT reagent in PBS, and the cells were incubated for 4 h at 37 °C. After removing the supernatant containing MTT solution, 100 μl of dimethyl sulfoxide (DMSO, Sigma-Aldrich) was added to dissolve the formazan. The optical density (OD) values at 595 nm were determined using a microplate reader (Thermo Fisher Scientific Inc., Waltham, MA, USA).

### Colony formation assay

Colony formation assay was performed to evaluate the survival fraction of cells with different treatments. Briefly, transfected cells were seeded in 6-well plates and irradiated at 4 Gy, followed by incubation for 10 days. The naturally formed colonies with more than 50 cells were fixed with 75% ethanol and stained with 0.5% (w/v) crystal violet solution (Sigma-Aldrich). The number of colonies was counted under a microscope (Leica Microsystems, Wetzlar, Germany).

### Flow cytometry analysis of cell apoptosis

Cell apoptosis was detected by the Annexin V/PI Apoptosis Detection Kit (KeyGEN Biotech, Nanjing, China) according to the manufacture’s instruction. In brief, approximately 5 × 10^5^ transfected cells irradiated with a dose of 4 Gy was harvested and re-suspended in 1 × Binding Buffer. Subsequently, cells were incubated with 5 μl Annexin V-FITC at room temperature for 15 min, followed by incubation with 10 μl propidium iodide (PI, 10 mg/ml) in the dark at room temperature for 5 min. Finally, the samples were analyzed using a FACScan flow cytometer (Becton–Dickinson; San Jose, CA, USA). Each sample was performed in triplicate.

### Caspase-3 activity assay

The activity of caspase-3 activity was detected using Caspase-3 assay kit (Beyotime Institute of Biotechnology) according to the protocol of manufacturer. Briefly, transfected cells after 4 Gy IR were collected and added into 10 μl of reaction buffer with the kit, followed by incubation for 2 h at 37 °C. Then the optical density value was measured at the wavelength of 400 nm. The relative caspase-3 activity of each sample was calculated as the absorbance of the well by that of the control group.

### Dual luciferase activity assay

Potential targets of miR-153-3p were searched with TargetScan (http://www.targetscan.org/), revealing that BCL2 is a potential target of miR-153-3p. Subsequently, dual luciferase activity assay was performed to verify whether BCL2 as a target of miR-153-3p. In brief, the human BCL2 3′UTR containing a putative miR-153-3p-binding site was cloned into the luciferase reporter vector psiCHECK-2 vector (Promega, Biotech Co., Ltd) to construct the wild-type-psiCHECK-2-BCL2-WT plasmid. The mutant-type luciferase vector psiCHECK-2-BCL2-MUT, harboring the mutant miR-153-3p binding site was also constructed. For cell transfection, HEK-293T cells were seeded in triplicate in 24-well plates and transfected with 100 ng of empty vector, psiCHECK-2-BCL2-WT, or psiCHECK-2-BCL2-MUT together with miR-153-3p or miR-NC using Lipofectamine 2000 (Invitrogen), followed by the detection of luciferase activity with a Dual Luciferase Reporter Assay kit (Promega). Each sample was performed in triplicate.

### Western blotting

Total proteins were extracted using RIPA buffer (Beyotime, Shanghai, China) and quantified using a BCA protein assay kit (Pierce). Equal amounts of proteins (50 g) were separated on a 10% sodium dodecyl sulfate–polyacrylamide gel and transferred to polyvinylidene fluoride membranes (PVDF, Millipore, Bedford, MA, USA). Then, the membranes were blocked with 5% nonfat milk in PBS-Tween-20 for 1 h and incubated with primary antibodies against BCL2 (1: 500, Abcam) or GAPDH (1: 5000, Cell Signaling Technology) at 4 °C overnight. Next, the membranes were incubated with horseradish peroxidase (HRP)-conjugated secondary antibody (1: 5000, Cell Signaling Technology) at room temperature for 2 h. The protein signals were visualized using an enhanced chemiluminescence (ECL) detection reagent (Pierce).

### Tumor formation in nude mice

The procedures of the animal experiments were approved by the Committee on the Use and Care of Animals of The First Hospital of Jilin University (Changchun, China). A total of 2 × 10^6^ U251 cells transfected with equivalent amount of miR-153-3p or miR-NC were subcutaneously injected into the left flank of 4- to 6-week-old BALB/c nude mice (n = 4 per group). The mice were exposed to a dose of 8 Gy when the tumor volume was about 100 mm^3^. Every 5 days after injection, the tumor volumes were measured with calipers and calculated according to the formula: tumor volume (mm^3^) = 0.5 × length × width^2^. On day 35 after injection, nude mice were sacrificed and the average tumor weight was measured. Meanwhile, xenograft tumors were rapidly removed and the expression of miR-153-3p or BCL2 was determined as the above described method. All animal experiments were conducted in strict accordance with the principles and procedures approved by the Committee on the Ethics of Animal Experiments of Liaoning Cancer Hospital & Institute (Liaoning, China).

### TUNEL staining

TUNEL assay was performed for detecting the apoptotic cells in xenograft tissues. In brief, paraffin cross-sectios (5-μm thick) of xenograft tissues were deparaffinized and rehydrated, followed by TUNEL staining using TUNEL Bright-Red Apoptosis Detection Kit (Vazyme) according to the manufacturer’s instructions. TUNEL-positive cells were observed and compared under fluorescence microscopy (DMI4000B, Leica).

### Statistical analysis

Statistical analysis was carried out using SPSS 13.0 statistical software and the data were expressed as mean ± standard error (SD) from at least three separate experiments. Student’s t-test and one-way analysis of variance (ANOVA) were used to analyses the significant differences between the two groups and among the groups, respectively. Pearson’s correlation test was used to analyze the linear correlation between miR-153-3p and BCL2 expression. The values of *p* < 0.05 were considered to be statistically significant.

## Results

### MiR-153-3p was down-regulated in radioresistant glioma tissues and cells in response to IR

**Fig. 1:**
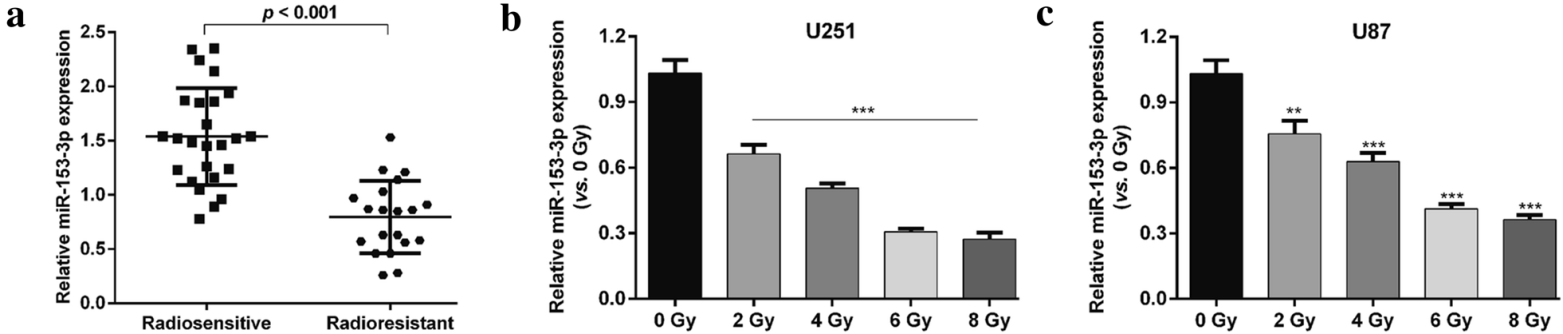
MiR-153-3p expression in radioresistant glioma tissues and cell lines treated with irradiation. **a** The expression profiles of miR-153-3p in radiosensitive (n = 25) and radioresistant (n = 20) glioma patient tumors; MiR-153-3p expression in **b** U251 cells and **c** U87 cells after irradiation treatment at different doses (0 Gy, 2 Gy, 4 Gy, 6 Gy and 8 Gy) by quantitative real-time PCR analysis. Data represent mean ± SD. **p < 0.01, ***p < 0.001 versus 0 Gy

MiR-153-3p has been reported to be a tumor suppressor, but whether it functions as a responder to IR remains unclear. To investigate this, we firstly determined the expression patterns of miR-153-3p in radiosensitive (n = 25) and radioresistant (n = 20) glioma patient tumors. As shown in Fig. 1a, miR-153-3p expression level was significantly down-regulated in radioresistant glioma tumors compared with that in radiosensitive tumors. In addition, the expression of miR-153-3p was also detected in U251 and U87 cells following exposure to increasing doses of IR. Consistently, miR-153-3p expression levels were markedly decreased dose dependently after IR treatment in U251 (Fig. 1b, *p* < 0.001) and U87 (Fig. 1c, *p* < 0.01, *p* < 0.001) cells. These data indicated that miR-153-3p might be associated with radiosensitivity in glioma.

### MiR-153-3p overexpression enhanced radiosensitivity of glioma in vitro

**Fig. 2:**
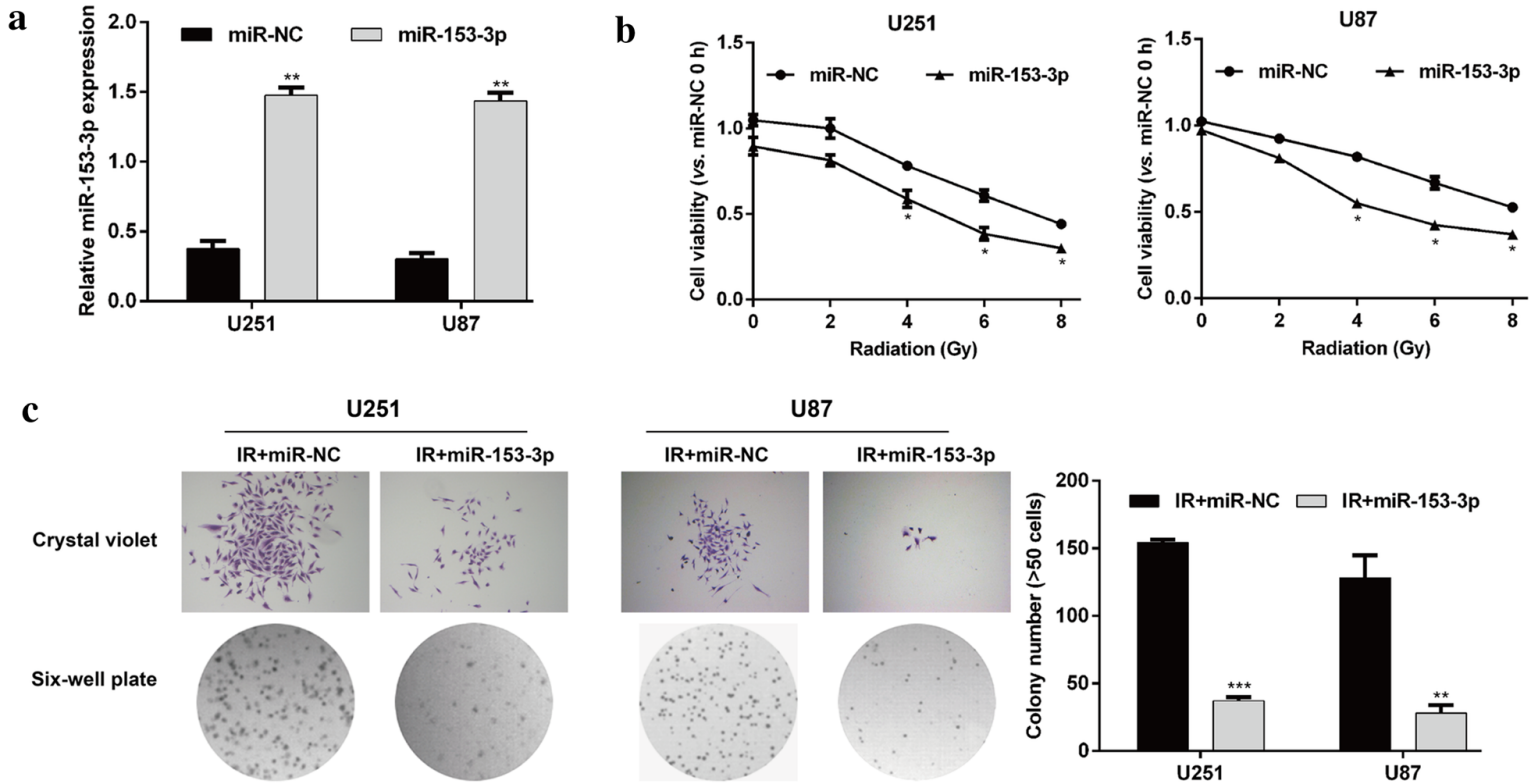
MiR-153-3p regulated glioma cell viability and proliferation after IR exposure. **a** The expression of miR-153-3p in U251 and U87 cells transfected with miR-153-3p or miR-NC was determined using quantitative real-time PCR. **b** Transfected U251 and U87 cells were exposed to different doses of IR, followed by cell viability evaluation by MTT assay. **c** The transfected U251 and U87 cells were treated with IR at dose of 4 Gy, followed by the measurement of colony formation. Data represent mean ± SD. *p < 0.05, **p < 0.01, ***p < 0.001 versus miR-NC or IR + miR-NC; IR: irradiation

**Fig. 3:**
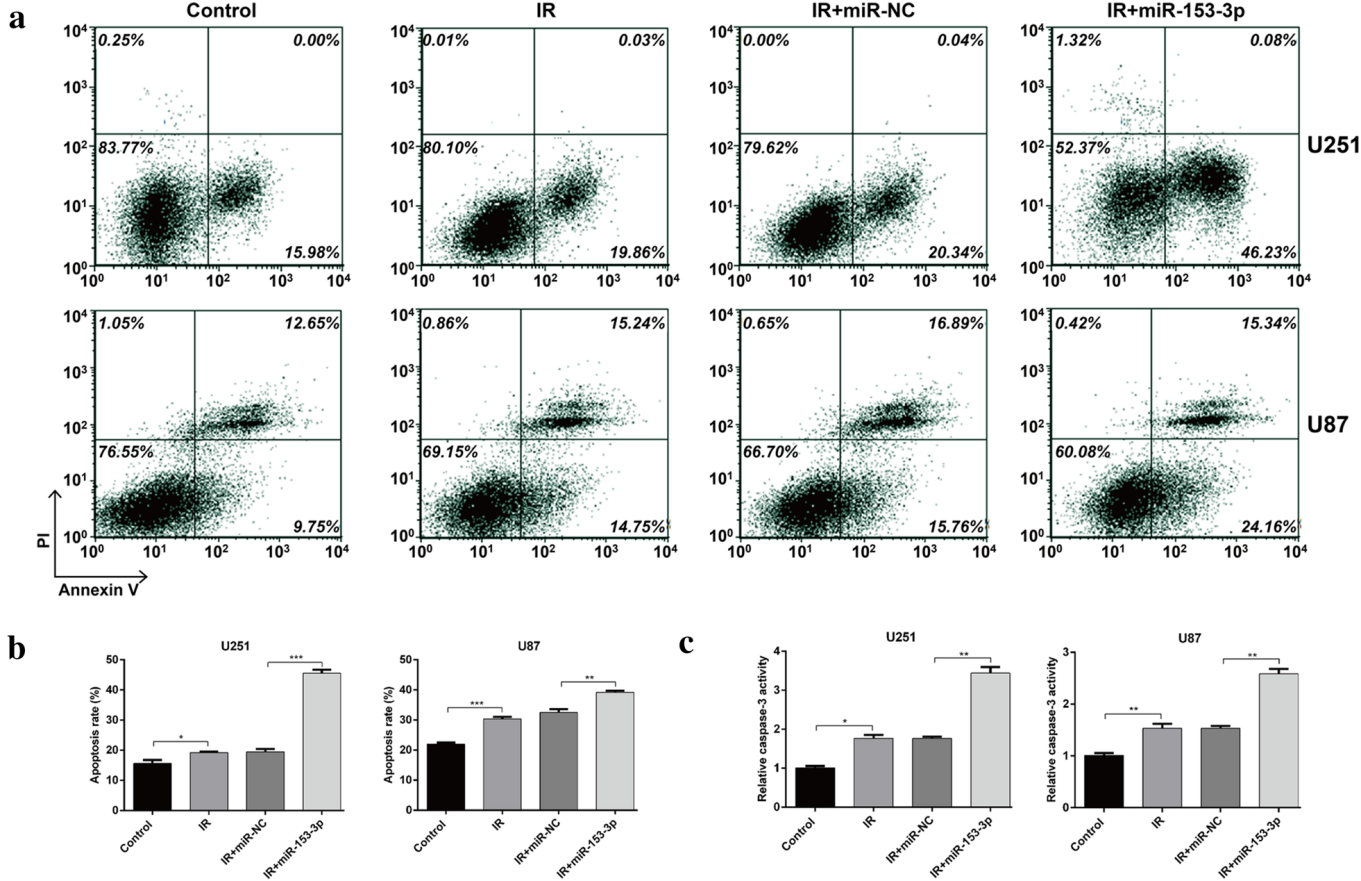
MiR-153-3p enhanced IR-mediated apoptosis in glioma cells. **a**, **b** U251 and U87 cells with miR-153-3p or miR-NC transfection were subjected to 4 Gy IR, followed by the measurement of cells apoptosis rate by flow cytometry analysis. **c** Caspase-3 activity was measured in transfected U251 and U87 cells after 4 Gy IR treatment. Data represent mean ± SD. *p < 0.05, **p < 0.01, ***p < 0.001

To explore the role of miR-153-3p in glioma cells following exposure to IR, U251 and U87 cells were transfected with miR-153-3p or miR-NC, followed by treatment with different dose of IR. As shown in Fig. 2a, qRT-PCR analysis confirmed the expression of miR-153-3p was significantly up-regulated in U251 and U87 cells after transfected with miR-153-3p, in comparison with those of cells transfected with miR-NC (*p* < 0.01). MTT assay was used to assess cell viability and the results showed that IR decreased the cell viability in a dose dependent manner. Notably, miR-153-3p overexpression significantly enhanced the loss of cell viability by increasing doses of IR in U251 and U87 cells (Fig. 2b, *p* < 0.05). We further evaluate the effects of miR-153-3p overexpression on cell proliferation in glioma cells under IR treatment. As presented in Fig. 2c, colony formation assay manifested that miR-153-3p overexpression resulted in a marked decrease of colony number in U251 (*p* < 0.001) and U87 (*p* < 0.01) cells after 4 Gy treatment. Moreover, flow cytometry analysis (Fig. 3a and b) showed that IR at a dose of 4 Gy obviously increased the apoptosis rate, and the effect was more pronounced in the miR-153-3p overexpression group of U251 (*p* < 0.05, *p* < 0.001) and U87 cells (*p* < 0.01, *p* < 0.001). To further confirm this conclusion, caspase-3 activity was also detected. As expected, the caspase-3 activity of U251 and U87 cells was remarkably increased following exposure to IR at dose of 4 Gy, which was dramatically reinforced by miR-153-3p overexpression (Fig. 3c, *p* < 0.05, *p* < 0.01). Collectively, these data revealed that miR-153-3p could enhance the sensitivity of glioma cells to IR treatment.

### MiR-153-3p targeted BCL2 in glioma cells

**Fig. 4:**
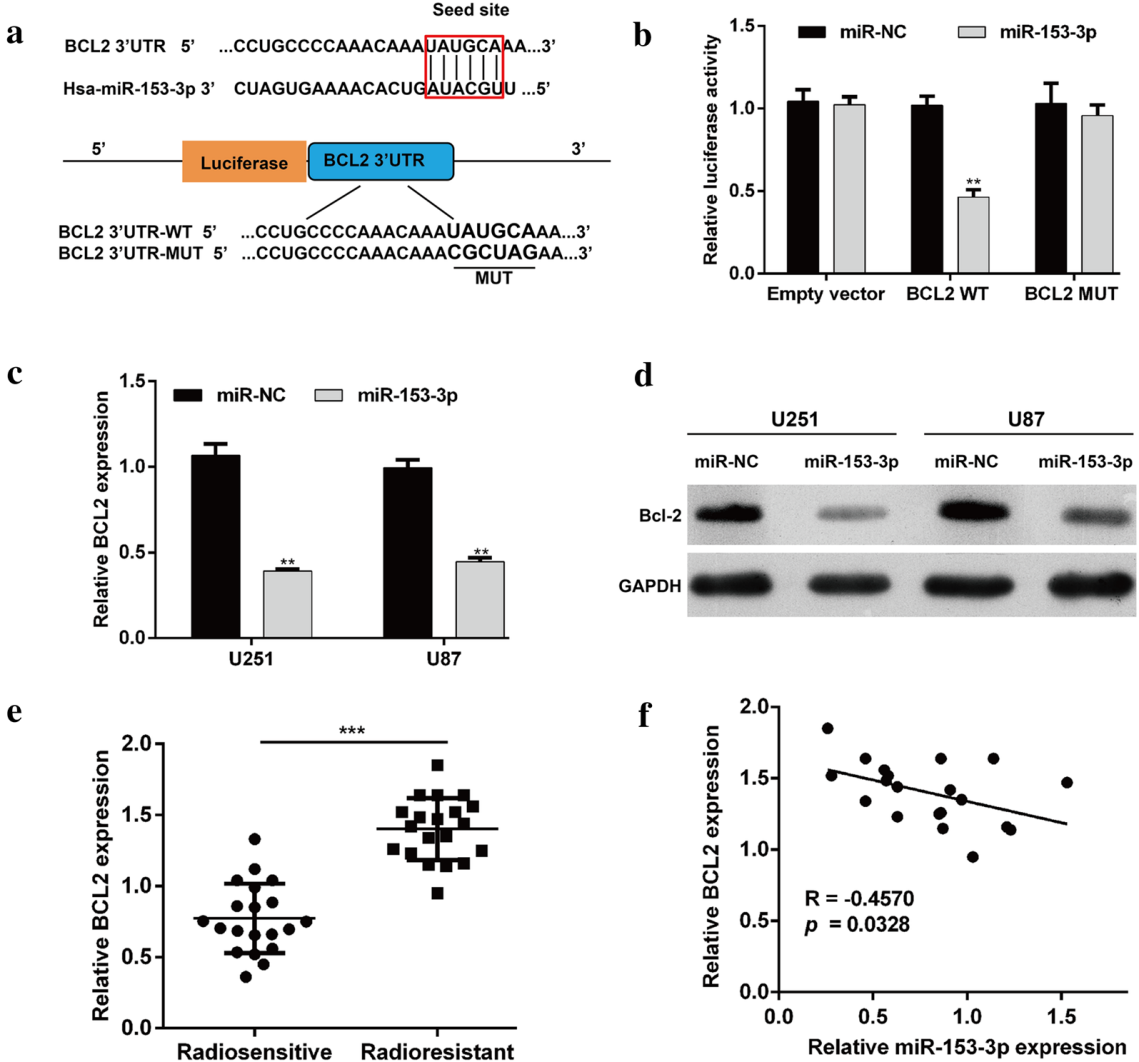
BCL2 was a downstream target of miR-153-3p in glioma cells. **a** The sequence alignment of human miR-153-3p with the 3′UTR of BCL2 is shown. The luciferase reporter constructs, BCL2 3′UTR-WT and BCL2 3′UTR-MUT were made using the seed sequence of miR-153-3p matching the 3′UTR of BCL2 mRNA. **b** HEK293 cells were co-transfected with the indicated plasmids and miR-153-3p or miR-NC for 48 h, and then they were subjected to the luciferase assay. U251 and U87 cells were transfected with miR-153-3p or miR-NC. The BCL2 mRNA (**c**) and protein (**d**) levels were determined by quantitative real-time PCR and Western blotting, respectively. Data represent mean ± SD. **p < 0.01 versus miR-NC; **e** the relative BCL2 mRNA levels in radioresistant glioma tumors (n = 20) and radiosensitive tumors (n = 25) were assessed using quantitative real-time PCR. ***p < 0.001 versus radiosensitive tumors; **f** correlation between the expression levels of BCL2 and miR-153-3p in radioresistant glioma tumors

To explore the underlying mechanism by which miR-153-3p enhances the effects of IR in glioma cells, an online database (TargetScan: http://www.targetscan.org/) was used to predict the possible targets of miR-153-3p. For its key role in regulating radiosensitivity of tumor cells [24, 25], BCL2 was selected as a potential target of miR-153-3p. As shown in Fig. 4a, the binding site of miR-153-3p was in the 3′UTR of BCL2 was presented. To validate this prediction, the dual luciferase reporter assay was performed in 293T cells. Results showed that luciferase activity in cells co-transfected with BCL-WT and miR-153-3p was strikingly decreased compared with controls, while showed no obvious changes in cells co-transfected with BCL-MUT and miR-153-3p (Fig. 4b, *p* < 0.01). Next, we determined the mRNA and protein levels of BCL2 in U251 and U87 cells after transfection with miR-153-3p or miR-NC. As shown in Fig. 4c, miR-153-3p overexpression resulted in a significant reduction of BCL2 mRNA in U251 and U87 cells (*p* < 0.01). Consistently, enforced expression of miR-153-3p obviously reduced the protein level of BCL2 in U251 and U87 cells (Fig. 4d). Further analysis indicated the expression of BCL2 mRNA in clinical samples revealed that the level of BCL2 mRNA was higher in radioresistant glioma tumors compared with that in radiosensitive tumors (Fig. 4e). Furthermore, the expression of BCL2 was inversely correlated with the miR-153-3p levels in radioresistant glioma tumors (Fig. 4f, *p* = 0.0328).

### Restoration of BCL2 alleviated miR-153-3p-mediated radiosensitivity of glioma cells

**Fig. 5:**
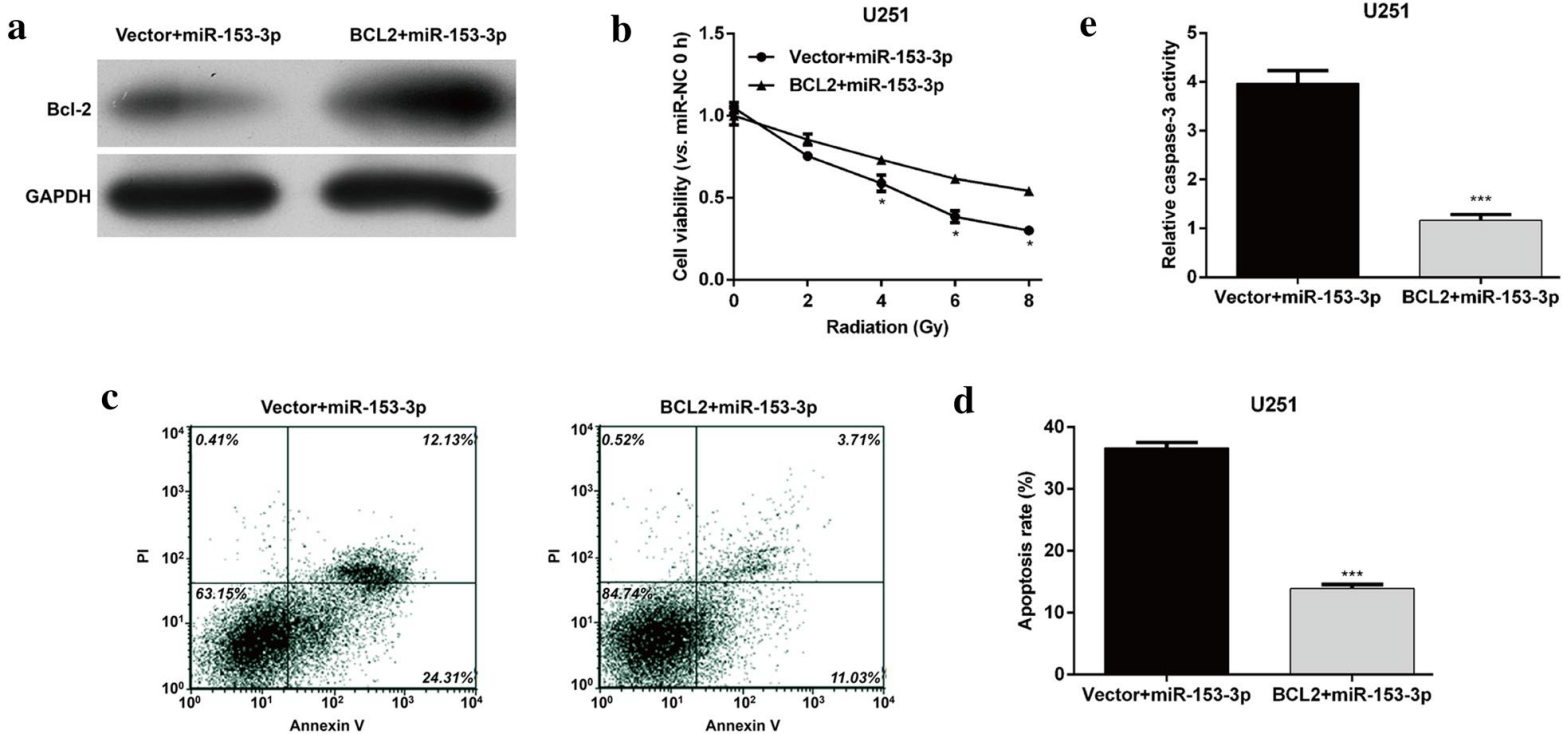
Restoration of BCL2 expression inhibited miR-153-3p-mediated radiosensitivity of glioma cells. U251 cells with miR-153-3p overexpression were transfected with empty vector or BCL2 vector. **a** The expression of BCL2 protein was determined using western blot analysis. **b** After exposure to different doses of IR, MTT assay was used to determine the cell viability. After exposure to 4 Gy IR, cell apoptosis and caspase-3 activity were detected using **c**, **d** flow cytometry analysis and **e** caspase-3 activity assay, respectively. Data represent mean ± SD. *p < 0.05, ***p < 0.001 versus Vector + miR-153-3p

As BCL2 was a downstream target of miR-153-3p, we speculated that miR-153-3p might enhance radiosensitivity of glioma cells through directly targeting BCL2. Thus, rescue experiments were performed to investigate the effect of BCL2 on the miR-153-3p-mediated radiosensitivity in glioma cells. First, western blotting analysis confirmed the expression of BCL2 protein levels was significantly increased in U251 cells co-transfected with miR-153-3p and BCL2 compared with miR-153-3p and empty vector co-transfection (Fig. 5a). MTT assay showed that restoration of BCL2 markedly weakened the inhibitory effect of miR-153-3p and IR exposure in a dose dependent manner (Fig. 5b, *p *< 0.05). Meanwhile, we further demonstrated that ectopic overexpression of BCL2 significantly attenuated the enhanced effects on apoptosis (Fig. 5c and d, *p *< 0.001) and caspase-3 activity (Fig. 5e, *p *< 0.001) conferred by miR-153-3p overexpression in U251 cells response to 4 Gy IR.

### MiR-153-3p enhanced glioma cell radiosensitivity in vivo

**Fig. 6:**
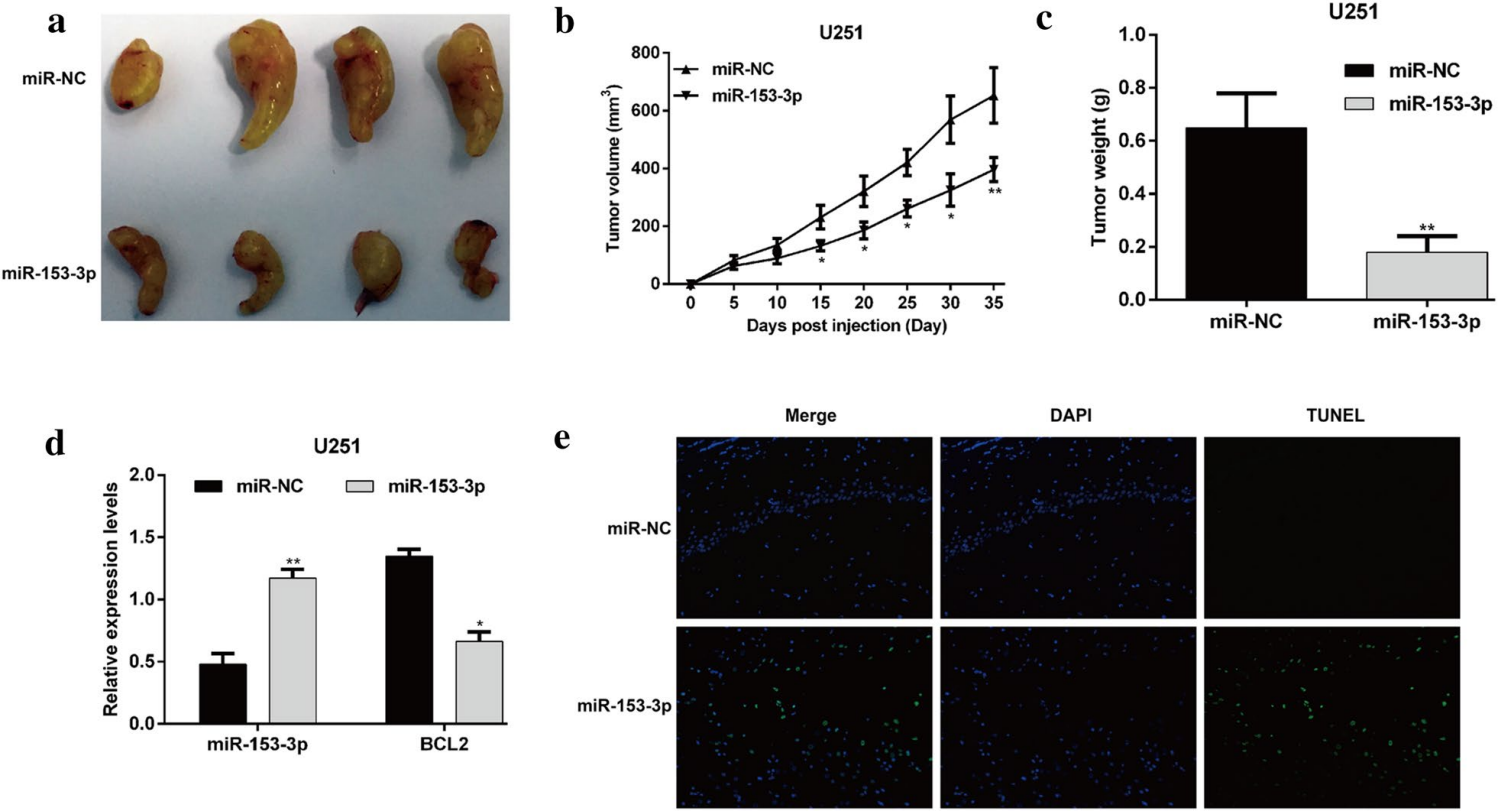
MiR-153-3p enhanced the tumor suppression capability of IR in vivo. U251/miR-NC or U251/miR-153-3p cells were subcutaneously injected into nude mice. The mice were exposed to IR at a dose of 8 Gy after 10 days injection. **a** Representative images of tumors formed in the mice in which miR-153-3p or miR-NC-infected U251 cells were implanted. **b** Tumor volumes were measured every 5 days after injection. **c** Tumors were dissected, and the tumor weights were measured on day 35 after inoculation. **d** Expression of miR-153-3p and BCL2 in miR-153-3p or miR-NC-expressing U251 cells-derived tumors by quantitative real-time PCR analysis. Data represent mean ± SD. **e** The TUNEL staining detected cell apoptosis in xenograft tumors. Blue staining represents the nucleus, and green staining indicates TUNEL-positive cells. *p < 0.05, **p < 0.01 versus miR-NC

To further study the inhibitory effect of miR-153-3p on glioma cell radiosensitivity in vivo, we injected U251/miR-NC or U251/miR-153-3p cells into nude mice. Radiation treatment with 8-Gy was initiated 10 days after injection. As shown in Fig. 6a, the size of the tumors derived from miR-153-3p-overexpressing U251 cells was significantly smaller than those from miR-NC group in a time course of 35 days. Moreover, the volume of the tumors was significantly suppressed in mice inoculated with miR-153-3p-overexpressing U251 cells compared with control groups (Fig. 6b, *p *< 0.05, *p *< 0.01). MiR-153-3p overexpression significantly decreased the tumor weights of the xenograft model mice treated with 8-Gy IR (Fig. 6c, *p *< 0.01). After 35 days, mice were sacrificed, and the tumors were collected to detect miR-153-3p and BCL2 expression. As shown in Fig. 6d, qRT-PCR analysis showed tumors derived from U251/miR-153-3p cells yielded significant increase of miR-153-3p, accompanied with decrease of BCL2, in comparison with tumors derived from U251/miR-NC cells. We also used TUNEL staining to detect cell apoptosis in xenograft tumors and found that miR-153-3p overexpression remarkably facilitated IR-induced apoptosis (Fig. 6e). These data again suggest that miR-153-3p overexpression increases the sensitization of glioma cells to IR-induced apoptosis.

## Discussion

Tumor cell proliferation and defective apoptosis pathway are considered as major mechanisms of radioresistance in cancer cells [26, 27]. In recent years, accumulating studies have suggested that many miRNAs are involved in radioresistance and oncogenesis of human malignancies, possibly through these mechanisms [28]. Here, we observed that miR-153-3p is down-regulated in radioresistant glioma tissues and cells exposure to IR. Furthermore, enhanced expression of miR-153-3p exaggerated glioma cells from the consequences of IR with the characteristics of inhibited cell viability, proliferation, and triggered apoptosis. The tumor volume and weight were also suppressed by miR-153-3p overexpression in xenograft model mice. Notably, BCL2 was predicted and confirmed as a direct target gene of miR-153-3p participating in the radiosensitivity in glioma.

According to the study from Patil K et al. miR-153-3p plays an important role in neuroprotection, which could down-regulate key proteins involved in Parkinson’s disease [29]. We thus speculated that miR-153-3p might be a tumor suppressor participating in the development of glioma. In fact, low-expression of miR-153-3p has been confirmed in several types of malignancies, and in general is correlated with less tumorigenicity and metastasis, but the evidence for its role in radioresistance has not been reported. In the present study, we found IR resistant glioma specimens exhibited lower levels of miR-153-3p. Considering miR-153-3p as a putative modulator of radiotherapy resistance, the regulation of miR-153-3p and/or its target BCL2 will help to explore radiotherapy sensitizing agents.

BCL2, an anti-apoptotic BCL2 family member, mainly locates on the outer member of mitochondria [30]. It is known that BCL2 plays crucial roles in the prevention of apoptosis in response to a variety of stimuli by blocking release of cytochrome c from mitochondria, and followed by inactivation of caspase cascades [31]. Previous research has revealed an elevation of BCL2 in cancerous cells exposed to IR, implying these cells have the potential to adapt to radiotherapy [22]. It is clear that the expression of BCL2 contributes to the malignant progression of radiation-resistant cancers [32]. For example, Wu et al. [33] observed that the combination of BCL2 antagonist ABT-737 and radiation-induced apoptosis resulted in decreased proliferation of breast cancer cells. Up-regulation of RACK1 confers resistance to radiation in esophageal cancer partially through up-regulating BCL2 [34]. In prostate cancer, BCL2/BAX ratio is involved in modulation of radiosensitivity [35].

Accumulating previous findings accompanied by the bioinformatic target predication made us analyze whether there is a crosstalk between miR-153-3p and BCL2. Our results demonstrated that miR-153-3p could directly target BCL2, and negatively regulate its protein levels. Transfection of BCL2 into miR-153-3p-overexpression cells was correlated with increased cell viability and survival, along with decreased caspase-3. Furthermore, down-regulation of BCL2 was observed in radiosensitive miR-153-3p-overexpressing glioma xenograft mice model. In line with previous studies implying the role of BCL2 in radiotherapy insensitivity, we thus suggest that miR-153-3p enhances radiation sensitivity of glioma cells through targeting BCL2. It is widely accepted that caspase-3 is the final executioner phase of apoptosis, indicating that miR-153-3p induces glioma cells apoptosis via BCL2/caspase-3 pathway.

## Conclusion

In summary, our results show that miR-153-3p overexpression elevated the sensitivity of glioma cells respond to IR in vitro and in vivo. The current study suggests a predictive role of miR-153-3p expression in radiotherapy management for glioma patients, and might shed new light on developing agents to enhance the antitumor effect of radiotherapy.
